# Terson Syndrome in Patients with Aneurysmal Subarachnoid Hemorrhage: A 10-Year Single-Center Experience

**DOI:** 10.1007/s12028-023-01701-9

**Published:** 2023-03-22

**Authors:** Jennifer Göttsche, Volker Knospe, Thomas Sauvigny, Nils Schweingruber, Jörn Grensemann, Martin S. Spitzer, Manfred Westphal, Christos Skevas, Patrick Czorlich

**Affiliations:** 1 Department of Neurosurgery, University Medical Center Hamburg-Eppendorf, Hamburg, Germany; 2 Department of Ophthalmology, University Medical Center Hamburg-Eppendorf, Hamburg, Germany; 3 Department of Neurology, University Medical Center Hamburg-Eppendorf, Hamburg, Germany; 4 Department of Intensive Care Medicine, University Medical Center Hamburg-Eppendorf, Hamburg, Germany

**Keywords:** Terson syndrome, Subarachnoid hemorrhage, Vitreous hemorrhage, Pars plana vitrectomy

## Abstract

**Background:**

Terson syndrome (TS), an intraocular hemorrhage associated with aneurysmal subarachnoid hemorrhage (aSAH), occurs in up to 46% of all patients with subarachnoid hemorrhage. Despite its high incidence, TS is underrepresented in the literature, and patients with aSAH are sometimes not systematically evaluated for the presence of TS in clinical practice. This work aims to raise awareness of TS, reevaluate previous scientific findings, describe risk factors associated with the occurrence of TS, and present our local diagnostic and treatment concept.

**Methods:**

All patients with aSAH treated at our institution between October 2010 and May 2020 were included in this retrospective study. The frequency of ophthalmological screening by indirect funduscopy, as well as the results, was investigated. In addition, the collection and statistical analysis of epidemiological and clinical data was performed using χ^2^, Kruskal–Wallis, and analysis of variance testing; multivariate regression; and receiver operating characteristic analysis. The significance level was set at *p* < 0.05.

**Results:**

A total of 617 patients were treated for aSAH in our institution. Of these, 367 patients (59.5%) were ophthalmologically examined for the presence of TS. The rate of TS in the examined patients was 21.3% (*n* = 78). Patients with TS had significantly higher Fisher and World Federation of Neurosurgical Societies (WFNS) scores (*p* < 0.0001). Regression analyses showed WFNS grade (*p* = 0.003) and the occurrence of seizures (*p* = 0.002) as independent predictors of TS, as did receiver operating characteristic analyses, which had a significant area under the curve of 0.66 for the combination of WFNS grade and seizures. For 12 (15.4%) patients, the TS had to be surgically treated by pars plana vitrectomy in a total of 14 eyes, which resulted in significant improvement of visual function in all patients: mean preoperative best-corrected visual acuity was 0.03 (± 0.08) versus 0.76 (± 0.21) postoperatively (*p* < 0.001).

**Conclusions:**

TS is a common complication in patients with aSAH, affecting approximately one in five patients. A higher WFNS grade and the occurrence of seizures are associated with TS; therefore, screening for TS should be performed in these patients.

## Introduction

Terson syndrome (TS), first described by Moritz Litten in 1881 and named after the French ophthalmologist Albert Terson, is a relatively common complication after subarachnoid hemorrhage (SAH), with rates of 10.2–46% in the literature [1–3]. It refers to an intraocular hemorrhage, for example, into the subhyaloid or intraretinal layers of the retina, or a vitreous hemorrhage (VH) which represents 3–5% of cases in the literature [2, 4, 5].

Spontaneous clearance of TS occurs frequently. In cases of failing spontaneous clearance, ophthalmological surgery by pars plana vitrectomy (PPV) should be considered and has been described as a safe intervention with an immediate improvement of visual acuity, as a significant acceleration of the process can be achieved surgically [6–8].

In addition to the significance for the surviving patient in terms of visual function, the presence of TS also appears to play a prognostic role even in the initial stage of SAH. Sung et al. were able to show an association with increased mortality in patients with SAH with TS [9]. Risk factors for the occurrence of this complication have not been widely studied. Lee et al. were able to identify the initial Word Federation of Neurosurgical Societies (WFNS) grade, aneurysm size, and treatment modality of the ruptured aneurysm as risk factors [3]. The initial loss of consciousness has also been suggested as a predictor for the occurrence of TS [10].

The aim of this study was to raise awareness of TS, reevaluate previous scientific findings, describe risk factors associated with the occurrence of TS, and present our local diagnostic and treatment concept.

## Methods

All 617 patients presenting to our intensive care unit with aneurysmal SAH (aSAH) between October 2010 and May 2020 were included in this study. SAH was confirmed by cranial computed tomography (cCT), magnetic resonance imaging, and/or lumbar puncture, as described before [11].

**Fig. 1:**
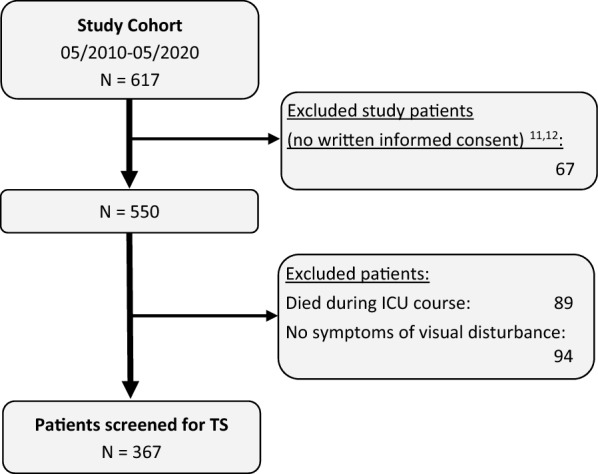
Flowchart. The flowchart shows patient exclusions. ICU intensive care unit, TS Terson syndrome

Patients were not screened for TS if they or their legal guardian did not consent to participate in our previous studies, if they died in-hospital, or were awake and fully oriented in the clinical investigations (and did not show any visual symptoms of TS, such as weakened or absent light reaction, subjective visual disturbances, or loss of red reflex) [11, 12]. In the end, 367 patients could be examined by an experienced neuro-ophthalmologist (VK and CS) by direct and indirect ophthalmoscopic examination after induced mydriasis (Tropicamid, Mydriaticum Stulln; Pharma Stulln GmbH, Stulln, Germany) prior to transfer to the rehabilitation unit or prior to discharge [11] (Fig. 1).

In the case of TS, necessary treatment was evaluated by the Department of Ophthalmology (CS) and determined according to the individual patient’s needs (watch and wait versus PPV), as described before [13]. In addition, best-corrected visual acuity (BCVA) was evaluated in awake patients scheduled for a PPV or watch and wait [14]. The perception of hand movement is stated with a BCVA of 0.005, whereas the ability to count fingers is stated with a BCVA of 0.014.

Patients demographics and aSAH-specific parameters were collected. The aneurysmal origin of SAH was proved by either digital subtraction angiography, cCT angiography, and/or magnetic resonance imaging angiography. These data included demographic information, aneurysm size and location, and distinct clinical evaluation scores (WFNS grading system, Fisher score). All events were assessed as seizures regardless of whether these occurred preclinically or during the inpatient course. If an epileptic seizure was observed outside the hospital, it was documented by a specialized emergency physician on-site and subsequently confirmed in the hospital by an Electroencephalography with epilepsy-typical potentials. Data were extracted from the intensive care unit’s electronic documentation system (Integrated Care Manager; Dräger Medical Deutschland GmbH, Lübeck, Germany).

Statistical analysis was performed using SPSS Statistics, Version 25 (IBM, Armonk, NY). Metric data are presented as the mean and standard deviation. Statistical analysis of the data was performed by a univariate analysis using χ^2^ tests, independent-samples Kruskal–Wallis tests, or analysis of variance tests, depending on the scale of the measurements and equality of variances. Logistic regression analyses were calculated to test the predictive power of clinical parameters previously described in the literature for the occurrence of TS. To evaluate the predictive performance of these predictors, receiver operating characteristic curves and corresponding areas under the curve (AUCs) were calculated. The statistical level of significance was set at *p* < 0.05.

This study was conducted according to the Declaration of Helsinki, as well as local and institutional laws, and was reported to the local ethical committee (Ethik-Kommission der Ärztekammer Hamburg, 2022-100874-WF). Parts of the data sets derive from the prospective studies with written consent of the patients and were anonymized after the studies were completed [11, 12]. Written informed consent was waived for this kind of study because of the anonymous recording and processing of data.

## Results

Between October 2010 and May 2020, 617 patients with aSAH were treated in our clinic. Of these, 367 were examined ophthalmologically for the presence of TS. The rate of TS among the examined patients was 21.3% (78 of 367).

On univariate analysis, patients with TS and those without TS differed significantly in terms of the severity of the aSAH (Table 1). Patients with TS had significantly higher Fisher and WFNS grades.

**Table 1:** Patient descriptives TS vs. no TS on univariate analyses

	TS (*n* = 78)	No TS (*n* = 289)	*p* value
Sex, No. (%)			0.433
Male	22 (28.2)	95 (32.9)	
Female	56 (71.8)	194 (67.1)	
Age, mean (SD)	52.8 (11.0)	55.0 (13.6)	0.196
Arterial hypertension, No. (%)	26 (33.3)	111 (38.4)	0.411
Nicotine abuse, No. (%)	22 (28.2)	68 (23.5)	0.394
Occurrence of seizure, No. (%)	29 (37.2)	43 (14.9)	< 0.001
WFNS grade, median (IQR)	4 (4)	1 (3)	< 0.001
Fisher grade, median (IQR)	4 (0)	4 (1)	< 0.001
Initial loss of consciousness, No. (%)	44 (56.4)	103 (35.6)	0.001
Parenchymal hemorrhage, No. (%)	36 (46.2)	78 (27.0)	0.001
Aneurysm diameter (mm), mean (SD)	7.5 (4.9)	7.0 (4.7)	0.435
Aneurysm location, No. (%)			0.180
Anterior	29 (37.2)	113 (39.1)	
MCA	11 (14.3)	68 (23.5)	
ICA + PCOM	24 (31.2)	63 (21.8)	
Posterior	14 (18.2)	45 (15.6)	
ICU stay (days), mean (SD)	22.4 (13.2)	20.3 (11.5)	0.178

*ICA* internal carotid artery, *ICU* intensive care unit, *IQR* interquartile range, *MCA* middle cerebral artery, *PCOM* posterior communicating artery, *SD* standard deviation, *TS* Terson syndrome, *WFNS* World Federation of Neurosurgical Societies

In the next step, logistic regression analyses were performed to assess the independent predictive value of parameters that were significant in univariate analyses. Because of the correlation of Fisher grade and parenchymal hemorrhage (Pearson’s *R* = 0.35; *p* < 0.001) and WFNS grade and initial unconsciousness (Pearson’s *R* = 0.51; *p* < 0.001), of these, only Fisher grade and WFNS grade were considered for the regression analyses.

Regression analyses found WFNS grade and the occurrence of seizures to be independent predictors for the occurrence of TS (Table 2).

**Table 2:** Logistic regression analyses

Tested variables	OR	CI	*p* value
Terson syndrome			
Fisher	1.448	0.980–2.141	0.063
WFNS	1.276	1.087–1.497	0.003
Seizure	2.528	1.405–4.550	0.002

*CI* confidence interval, *OR* odds ratio, *WFNS* World Federation of Neurosurgical Societies

**Fig. 2:**
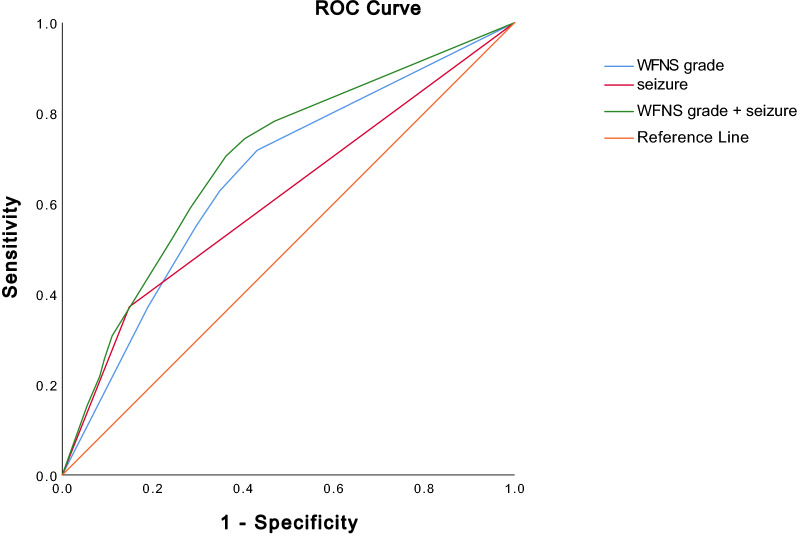
Receiver operating characteristic (ROC) curve. In this ROC curve, models for Terson syndrome prediction detected via multivariate logistic regression were displayed. Models were tested for correct outcome prediction against the null hypothesis. WFNS World Federation of Neurosurgical Societies

Receiver operating characteristic curves were calculated for the occurrence of TS using the significant parameters from regression analysis (Fig. 2). The AUC for WFNS grade was 0.66 (confidence interval [CI] 0.59–0.73; *p* < 0.001). The AUC for seizures was 0.61 (CI 0.54–0.69; *p* = 0.002). Combining both parameters resulted in an AUC of 0.70 (CI 0.63–0.76).

Furthermore, the patients with TS were examined for the necessity of surgical treatment and its outcome. There were 12 (15.4%) patients who had a total of 14 eyes treated surgically by PPV for TS. Postoperatively, visual function improved, as shown in Table 3. BCVA improved significantly in all patients at a postoperative mean of 0.76 (± 0.21), compared to 0.03 (± 0.08) preoperatively (*p* < 0.001). Surgeries were performed 6–8 weeks after the initial hemorrhage. No internal limiting membrane peeling was performed because no internal limiting membrane bleeds were present.

**Table 3:** Preoperative and postoperative visual function in patients with vitrectomy [14]

Patient no	Affected eye	Visual function preoperatively	Visual function postoperatively
1	Left	0.005	0.8
1	Right	0.005	0.8
2	Left	0.005	0.8
3	Right	0.005	0.8
4	Right	0.014	0.7
5	Right	0.005	1.0
6	Right	0.014	1.0
7	Right	0.014	1.0
8	Right	0.005	0.5
9	Right	0.005	0.5
10	Right	0.3	0.5
11	Right	0.005	1.0
12	Left	0.005	0.5
12	Right	0.005	0.8

## Discussion

TS is a long-known complication of aSAH and usually affects about every fifth patient, consistent with the 21.3% reported in this large cohort. It is even more astonishing that, on the one hand, patients in many hospitals are still not systematically examined for the presence of TS; on the other hand, there are still large gaps in the literature. The aim of this work is to bring TS back into the focus of neurosurgeons, neurologists, ophthalmologists, and intensive care physicians and to share our 10-year experiences.

The gold standard for the screening of TS is direct and indirect funduscopy by an ophthalmologist [11, 15]. However, various studies have shown that TS can also be diagnosed using ocular ultrasound (OU) and cCT [12, 16–20]. In one of our previous studies we found that sensitivity and specificity values for the detection of vitreous hemorrhage via OU increased with the investigator’s number of examinations up to 81.8% and 100%, respectively [12]. Galán et al. report the sensitivity and specificity of OU to be 87.5% and 98.5%, respectively, for the detection of vitreous hemorrhage [16]. Koskela et al. report a sensitivity of 42% for computed tomography findings regarding TS and a specificity of 97% [20].

Further diagnostic modalities were evaluated by Ramos-Estebanez et al. [15], showing the feasibility of bedside optic coherence tomography examination in patients with aSAH. The use of ultrasound and computed tomography provides increased the sensitivity and specificity, especially in the diagnosis of vitreous hemorrhage, making these methods particularly useful for identifying patients who should be treated surgically. Because ultrasound diagnosis in particular is ubiquitous in the intensive care unit, it is also suitable for hospitals in countries with limited resources or a shortage of ophthalmologists because it is easy to learn and can be performed quickly [12].

The question now arises as to which patients should be screened for TS, particularly in hospitals and health care systems with limited personnel and financial resources.

Several factors have been described in the literature to be associated with the occurrence of TS, such as high Hunt and Hess and WFNS grades, low Glasgow Coma Scale scores and high Fisher scale scores [3, 21–25]. An initial loss of consciousness and an initially raised intracranial pressure (ICP) have also been investigated as predictors for TS [10, 26]. In the work of Joswig et al. [26], all patients with TS presented with pathological ICP values of > 20 cm H_2_O.

These results partially align with this study’s results, as we were also able to show the association of a high WFNS grade with the occurrence of TS in our collective. We were also able to demonstrate an association between seizures and the occurrence of TS. In recent years, the literature has shown that seizures are associated with the severity of SAH and neuroinflammation and that these factors are also related to the possible pathomechanisms of TS [27, 28].

Because the pathophysiology of TS has not yet been conclusively clarified, a direct pathophysiological explanation for the occurrence of TS and an increased occurrence of seizures is missing [10, 29]. The favored pathomechanism in the literature is intraorbital venous congestion due to increased ICP and subsequent rupture of the small retinal vessels [29]. The increase in ICP certainly predisposes to the development of TS, yet not all patients with TS have been shown to have an increase, implying additional underlying pathomechanisms [10]. Other explanations are that intracranial blood penetrates directly into the vitreous cavity through the lamina cribrosa, the peripapillary and perivascular leak theory, and finally a primary vitreous origin [29–33].

In our opinion, different pathomechanisms for the development of TS should be suspected, as an increase in bleeding can be seen more than 14 days after SAH ictus, as shown previously by Vanderlinden et al. and by our group [5, 11].

For this reason, we screen for TS before patients are transferred to the rehabilitation units or before they are discharged. On the one hand, we are convinced of the reduced rate of overlooked TS with later development; on the other hand, however, there is the possibility of carrying out an ophthalmological treatment concept or PPV, if necessary.

The ideal point in time to perform a PPV is discussed controversially in the literature, as minor hemorrhages, as well as retinal hemorrhages, usually resolve spontaneously. Dense vitreous hemorrhages, on the other hand, lead to a loss of vision or relevant reduction of BCVA, which can also recover after a later PPV, but it seems obvious, even without evidence from studies, that an early improvement in BCVA should be aimed for because patients can then also take part in the rehabilitation programs in a more targeted and active manner [34]. Larger series have demonstrated that an early PPV can be performed safely and results in an immediate and significant improvement in vision [7, 13, 33, 35].

Limitations of the study are its partly retrospective character and the fact that not all patients with aSAH were screened for the presence of TS during the mentioned time period. After we conducted our prospective studies, patients who presented with a higher WFNS grade (III–V) and/or had neurological deficits before discharge or complained about subjective visual impairments were referred for an ophthalmological examination [11, 12]. Patients without any symptoms were not regularly examined by an ophthalmologist, as there was usually no need for treatment, even in the unlikely event that TS was detected, as described above, in which only minor hemorrhages were expected. Nonetheless, we were able to investigate a substantial number of TS cases, resulting in one of the largest TS cohorts published so far.

**Fig. 3:**
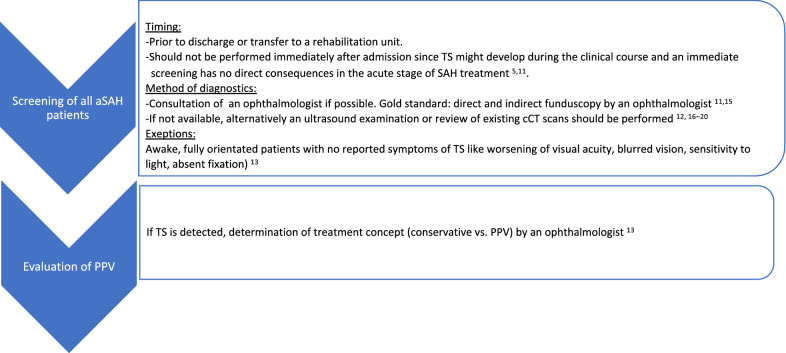
Recommendation for the approach of patients with aneurysmal subarachnoid hemorrhage (aSAH) regarding potential Terson syndrome (TS). The figure shows the approach of screening and potential treatment of TS regarding optimal timing and methods. cCT cranial computed tomography, PPV pars plana vitrectomy, SAH subarachnoid hemorrhage

Summarizing the existing literature and the 10-year experience of our facility, we recommend the approach shown in Fig. 3.

## Conclusions

TS is a common complication of aSAH, affecting approximately one in five patients. Because of the associations shown between higher WFNS grades and the occurrence of seizures and TS we suggest consistent screening in, but not limited to, these particular patients.
